# Exogenous γ-Aminobutyric Acid Can Improve Seed Germination and Seedling Growth of Two Cotton Cultivars under Salt Stress

**DOI:** 10.3390/plants13010082

**Published:** 2023-12-26

**Authors:** Zhiduo Dong, Jian Huang, Tong Qi, Ajing Meng, Qiuping Fu, Yanbo Fu, Fei Xu

**Affiliations:** 1 College of Water Conservancy and Civil Engineering, Xinjiang Agricultural University, Urumqi 830052, China; dzd1281228561@163.com; 2 Institute of Soil Fertilizer, Agricultural Water Saving, Xinjiang Academy of Agricultural Sciences, Urumqi 830091, China; huangjian@xaas.ac.cn (J.H.); maj19890917@163.com (A.M.); fuyanbo2010@163.com (Y.F.); xufei@xaas.ac.cn (F.X.); 3 Key Laboratory of Saline-Alkali Soil Improvement and Utilization (Saline-Alkali Land in Arid and Semi-Arid Regions), Ministry of Agriculture and Rural Affairs, Urumqi 830091, China; 4 College of Land Science and Technology, China Agricultural University, Beijing 100193, China; 5 National Soil Quality Aksu Observation Experimental Station, Aksu 843000, China

**Keywords:** ion content, *Gossypium*, osmoregulation, oxidative stress

## Abstract

Excessive salt content in soil has adverse effects on cotton production, especially during the germination and seedling stages. γ-aminobutyric acid (GABA) is an important active substance that is expected to improve the resistance of plants to abiotic stresses. This study focused on two cotton cultivars (*Gossypium hirsutum* L.: Tahe 2 and Xinluzhong 62) and investigated the impact of exogenous GABA (0, 1, 2, 3, and 4 mM) on seed germination, seedling growth, and related morphological, physiological, and biochemical indicators under salt stress (150 mM NaCl). The results showed that salt stress significantly reduced the germination rate and germination index of cotton seeds (decreased by 20.34% and 32.14% for Tahe 2 and Xinluzhong 62, respectively), leading to decreased seedling height and biomass and causing leaf yellowing. Salt stress induced osmotic stress in seedlings, resulting in ion imbalance (marked reduction in K^+^/Na^+^ ratio) and oxidative damage. Under salt stress conditions, exogenous GABA increased the germination rate (increased by 10.64~23.40% and 2.63~31.58% for Tahe 2 and Xinluzhong 62, respectively) and germination index of cotton seeds, as well as plant height and biomass. GABA treatment improved leaf yellowing. Exogenous GABA treatment increased the content of proline and soluble sugars, with varying effects on betaine. Exogenous GABA treatment reduced the Na^+^ content in seedlings, increased the K^+^ content, and increased the K^+^/Na^+^ ratio (increased by 20.44~28.08% and 29.54~76.33% for Tahe 2 and Xinluzhong 62, respectively). Exogenous GABA treatment enhanced the activities of superoxide dismutase and peroxidase, and reduced the accumulation of hydrogen peroxide and malondialdehyde, but had a negative impact on catalase activity. In conclusion, exogenous GABA effectively improved cotton seed germination. By regulating osmoprotectant levels, maintaining ion homeostasis, and alleviating oxidative stress, GABA mitigated the adverse effects of salt stress on cotton seedling growth.

## 1. Introduction

Soil salinization is a global environmental issue, affecting 20% of agricultural land and 33% of irrigated agricultural land, resulting in reduced crop yields and diminished arable land [1]. This phenomenon is particularly prevalent in arid and semi-arid regions, posing a significant challenge for agricultural productivity. Among the various salts affecting soil, NaCl stands out as the most common and widely distributed [1,2,3]. In this environmental context, cotton (*Gossypium* spp.), as a moderately salt-tolerant crop and a globally significant economic crop, plays a crucial role in the agricultural economy of regions affected by soil salinization [4,5,6,7,8]. However, excessively high salt levels in the soil have varying degrees of negative effects on the entire growth period of cotton, especially during the germination and seedling stages, where cotton is more sensitive to salt stress [4,8]. Salt inhibits the absorption of water and nutrients by cotton seeds and seedling roots, leading to the uptake of large amounts of salts, such as Na^+^ and Cl^−^, resulting in adverse effects such as osmotic stress, ion toxicity, and oxidative stress [3,8]. This poses the challenge of ‘difficult emergence and seedling protection’ when cultivating cotton in saline–alkali soil. In order to increase cotton yield, urgent measures are needed to enhance the germination capacity of cotton seeds under salt stress and improve the resilience of seedlings to adversity.

The treatment with exogenous substances stands out as an effective strategy for enhancing plant stress resistance [1,4,9]. Among these substances, γ-aminobutyric acid (GABA) emerges as an important active substance with the potential to improve plant resistance to non-biological stress. GABA, known for its environmentally friendly characteristics, actively participates in various physiological activities of plants, encompassing growth, development, signal transduction, and stress response [10,11,12]. There is substantial evidence indicating that exogenous GABA can improve seed germination, plant growth and development, and enhance plant tolerance to non-biological stress, such as salt, drought, low temperature, hypoxia, and heavy metals [9,10,11,12,13]. Additionally, GABA contributes to increased nutrient absorption and utilization efficiency by plants, such as nitrogen [9,10,14,15], phosphorus [16], potassium [17], and calcium [11,12,17]. Exogenous GABA also improves plant growth and alleviates the adverse effects of stress by enhancing antioxidant defense mechanisms [10,11,14,15]. Exogenous GABA induces an increase in the GABA content in plants [9,13], upregulates the tricarboxylic acid cycle to generate more energy, and plays a critical role in maintaining the carbon–nitrogen balance of plants [9,11,16]. Especially under salt stress, the accumulation of GABA can activate H^+^-ATPase, maintaining intracellular pH balance, thereby improving plant salt tolerance [11,13]. Recent studies suggest that foliar application of GABA has the potential to alleviate salt damage in cotton [17], highlighting its promising role in enhancing salt tolerance.

Existing research indicates that exogenous GABA has a positive impact on improving seed viability, germination performance, and seedling establishment under salt stress in various plants [12,18,19,20]. However, there is relatively limited information on the effects of GABA on improving cotton seed germination and seedling growth. *Gossypium hirsutum* L. is the most important cultivar in global cotton production, cultivated in over 95% of cotton-producing regions worldwide [3,4]. The Xinjiang region holds a significant position in the global cotton industry, especially in southern Xinjiang, where cotton cultivation covers more than two-thirds of the total cotton area in Xinjiang [6,7,21]. This study selected the main cotton cultivars, ‘Tahe 2’ and ‘Xinluzhong 62’, from southern Xinjiang, China [22]. The objectives were (1) to investigate the effects of high salinity (150 mM NaCl) stress [8] on the growth, biochemistry, and physiological adaptation mechanisms of these two cotton (*Gossypium hirsutum* L.) cultivars, aiming to understand the importance of variety-specific responses in comprehending the physiological and biochemical mechanisms under salt stress; (2) to systematically explore the impact of exogenous GABA at different concentrations on cotton seedlings under high salt (150 mM NaCl) stress, determining the appropriate GABA concentration and providing more specific application references; (3) to not only focus on seed germination and seedling growth but also delve into the changes in relevant morphological, physiological, and biochemical indicators. This comprehensive approach contributes to a thorough understanding of the impact of salt stress on cotton growth and reveals the mechanisms of exogenous GABA during this process. Specifically, we measured cotton seed germination rate and germination index, as well as plant height, biomass, osmoregulatory substances, ion content, reactive oxygen species metabolism products, and antioxidant enzyme activity. We anticipate that the study results will enhance the germination and salt tolerance of cotton seeds under salt stress, providing valuable insights for increasing cotton yield in saline–alkali soil.

## 2. Results

### 2.1. Germination Rate and Index of Germination

**Figure 1:**
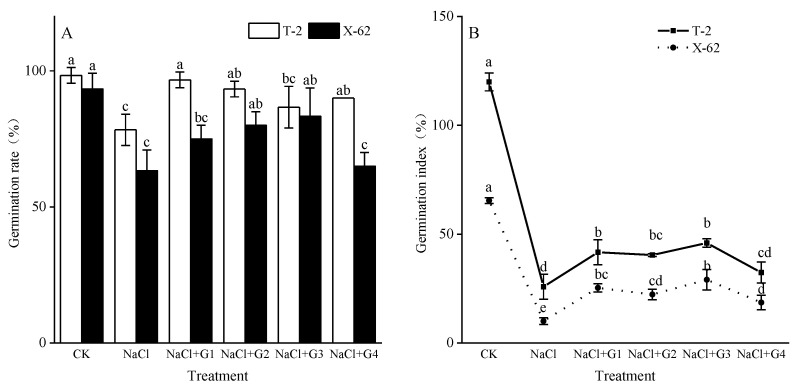
Effects of GABA on the germination rate (**A**) and germination index (**B**) of cotton seeds under salt stress. Different lowercase letters indicate a significant difference in the mean value of different treatments for the same cotton cultivar at the probability level of 0.05 (*p* < 0.05), determined by one-way analysis of variance (ANOVA) and Duncan’s post hoc test. The data are presented as means ± standard deviation (SD) calculated from three repetitions. T-2: Tahe 2; X-62: Xinluzhong 62. CK: 0 mM NaCl + 0 mM GABA; NaCl: 150 mM NaCl; NaCl + G1: 150 mM NaCl+ 1 mM GABA; NaCl + G2: 150 mM NaCl + 2 mM GABA; NaCl + G3: 150 mM NaCl + 3 mM GABA; NaCl + G4: 150 mM NaCl + 4 mM GABA.

The germination rate (GR) of cotton seeds for Tahe 2 (T-2) and Xinluzhong 62 (X-62) under the CK treatment were 98.33% and 93.33%, respectively (Figure 1A). Compared to CK, the GR of T-2 and X-62 significantly decreased under NaCl treatment (*p* < 0.05), with reductions of 20.34% and 32.14%, respectively. In comparison to the NaCl treatment, under NaCl + G1~G4 treatments, the GR of T-2 and X-62 showed varying degrees of increase. For T-2, the GR were significantly different from the NaCl treatment (*p* < 0.05), with increases ranging from 10.64% to 23.40%, and NaCl + G1 treatment exhibited the largest increase. For X-62, the GR increased by 2.63% to 31.58%, with NaCl+G3 treatment showing the largest increase.

The germination index (GI) of cotton seeds for Tahe 2 (T-2) and Xinluzhong 62 (X-62) under the CK treatment were 119.92% and 65.40%, respectively (Figure 1B). Compared to CK, the GI of T-2 and X-62 significantly decreased under NaCl treatment (*p* < 0.05), with reductions of 78.45% and 84.62%, respectively. In comparison to the NaCl treatment, the GI of T-2 and X-62 significantly increased under NaCl + G1~G4 treatments (*p* < 0.05). Among them, NaCl + G3 treatment exhibited the highest GI, with T-2 and X-62 showing rates 1.78 times and 2.89 times higher, respectively, compared to the NaCl treatment.

### 2.2. Seedling Height and Biomass

According to Table 1, NaCl treatment reduced the seedling height, aerial part fresh weight, aerial part dry weight, root fresh weight, total fresh weight, and total dry weight of both Tahe 2 (T-2) and Xinluzhong 62 (X-62) seedlings, while increasing the root dry weight. Compared to CK, under NaCl treatment, T-2 exhibited reductions in plant height, aerial part fresh weight, aerial part dry weight, root fresh weight, total fresh weight, and total dry weight by 10.10%, 25.68%, 20.84%, 22.43%, 24.02%, and 13.68%, respectively, with an increase of 2.50% in root dry weight. X-62 showed reductions in plant height, aerial part fresh weight, aerial part dry weight, root fresh weight, total fresh weight, and total dry weight by 24.89%, 21.93%, 19.43%, 24.53%, 23.22%, and 7.18%, respectively, with an increase of 19.47% in root dry weight. In comparison to NaCl treatment, NaCl + G1~G4 treatments increased the plant height, aerial part fresh weight, aerial part dry weight, root fresh weight, root dry weight, total fresh weight, and total dry weight of T-2 by 2.81~13.48%, 27.69~42.48%, 13.65~32.28%, 30.02~42.52%, 10.99~24.89%, 28.91~41.45%, and 14.91~29.45%, respectively. For X-62, NaCl + G1~G4 treatments increased plant height, aerial part fresh weight, aerial part dry weight, root fresh weight, total fresh weight, and total dry weight by 7.87~30.90%, 25.56~27.80%, 40.36~48.53%, 28.22~38.32%, 28~31.79%, 21.74~30.72%, respectively. However, only NaCl + G3 treatment slightly increased the root dry weight of X-62 compared to NaCl treatment, with an increase of 4.46%, while the other treatments showed a slight decrease in root dry weight ranging from 3.34% to 7.66%.

**Figure 2:**
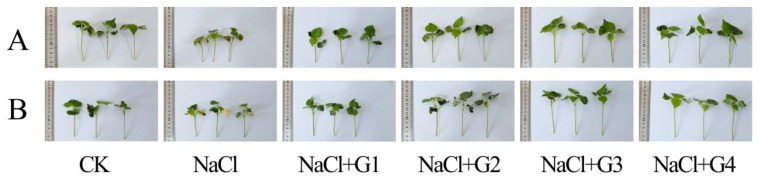
Phenotypes of cotton seedlings of Tahe 2 (**A**) and Xinluzhong 62 (**B**) grown for 31 d. CK: 0 mM NaCl + 0 mM GABA; NaCl: 150 mM NaCl; NaCl + G1: 150 mM NaCl + 1 mM GABA; NaCl + G2: 150 mM NaCl + 2 mM GABA; NaCl + G3: 150 mM NaCl + 3 mM GABA; NaCl + G4: 150 mM NaCl + 4 mM GABA.

Phenotypic observations and photographic records were made for Tahe 2 (T-2) (Figure 2A) and Xinluzhong 62 (X-62) (Figure 2B) seedlings under each treatment for 31 d. The results showed that, compared to CK, seedlings of T-2 and X-62 under NaCl treatment exhibited stunted plant growth, reduced leaf area, and yellowing of leaves. In contrast, under NaCl + G1~G4 treatments, cotton seedlings exhibited robust growth with no observed leaf yellowing. Additionally, plant height and leaf area were larger than those of seedlings under NaCl treatment. Phenotypic identification results indicated that cotton seedlings under NaCl + G1~G4 treatments displayed a favorable salt-tolerant phenotype. This observation provides the basis for analyzing the induced physiological and biochemical mechanisms underlying salt tolerance.

### 2.3. Osmoregulatory Protective Agent Levels

**Figure 3:**
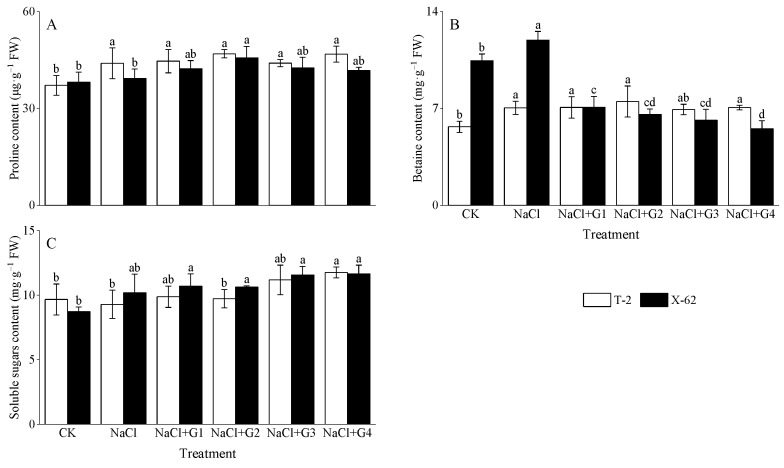
Effect of GABA on proline (**A**), betaine (**B**), and soluble sugars (**C**) in leaves of cotton seedlings under salt stress. Different lowercase letters indicate a significant difference in the mean value of different treatments for the same cotton cultivar at the probability level of 0.05 (*p* < 0.05), determined by one-way analysis of variance (ANOVA) and Duncan’s post hoc test. The data are presented as means ± standard deviation (SD) calculated from three repetitions. T-2: Tahe 2; X-62: Xinluzhong 62. CK: 0 mM NaCl + 0 mM GABA; NaCl: 150 mM NaCl; NaCl + G1: 150 mM NaCl + 1 mM GABA; NaCl + G2: 150 mM NaCl + 2 mM GABA; NaCl + G3: 150 mM NaCl + 3 mM GABA; NaCl + G4: 150 mM NaCl + 4 mM GABA.

NaCl treatment increased the levels of proline and betaine in the leaves of Tahe 2 (T-2) and Xinluzhong 62 (X-62) seedlings (Figure 3A,B). Compared to CK, under NaCl treatment, the proline content in T-2 and X-62 increased by 18.36% and 2.98%, respectively, with T-2 showing a significant difference from the CK treatment (*p* < 0.05). The betaine content in T-2 and X-62 increased by 24.11% and 14.23%, respectively, both significantly differing from the CK (*p* < 0.05). In comparison to NaCl treatment, NaCl + G1~G4 treatments increased the proline content in T-2 and X-62 by 0.12~6.73% and 6.16~16.11%, respectively, with NaCl + G2 treatment showing the greatest increase. For betaine content, compared to NaCl treatment, NaCl + G3 treatment slightly decreased, with a reduction of 1.72%, while the remaining treatments increased by 0.27~6.43%, with NaCl + G2 showing the highest increase. NaCl + G1~G4 treatments significantly decreased the betaine content in X-62 by 40.68~56.66% (*p* < 0.05), with NaCl + G4 treatment showing the greatest reduction.

From Figure 3C, it is evident that NaCl treatment had different effects on the soluble sugar content in the leaves of T-2 and X-62 seedlings. Compared to CK, NaCl treatment decreased the soluble sugar content in T-2 by 3.94%, while it increased the soluble sugar content in X-62 by 16.60%. In comparison to NaCl treatment, NaCl + G1~G4 treatments increased the soluble sugar content in T-2 and X-62 by 4.76~26.57% and 4.29~14.42%, respectively, with NaCl + G4 treatment showing the greatest increase.

### 2.4. Na^+^ Content, K^+^ Content, and the K^+^/Na^+^ Ratio

**Figure 4:**
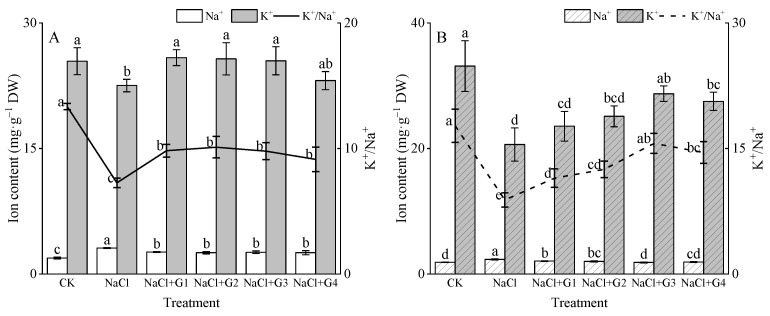
Effect of GABA on Na^+^ content, K^+^ content, and K^+^/Na^+^ homeostasis of Tahe 2 (**A**) and Xinluzhong 62 (**B**) cotton seedlings under salt stress. Different lowercase letters indicate a significant difference in the mean value of different treatments for the same cotton cultivar at the probability level of 0.05 (*p* < 0.05), determined by one-way analysis of variance (ANOVA) and Duncan’s post hoc test. The data are presented as means ± standard deviation (SD) calculated from three repetitions. T-2: Tahe 2; X-62: Xinluzhong 62. CK: 0 mM NaCl + 0 mM GABA; NaCl: 150 mM NaCl; NaCl + G1: 150 mM NaCl+ 1 mM GABA; NaCl + G2: 150 mM NaCl + 2 mM GABA; NaCl + G3: 150 mM NaCl + 3 mM GABA; NaCl + G4: 150 mM NaCl + 4 mM GABA.

From Figure 4A,B, it can be observed that NaCl treatment significantly increased the Na^+^ content in Tahe 2 (T-2) and Xinluzhong 62 (X-62) seedlings (*p* < 0.05). Compared to CK, under NaCl treatment, the Na^+^ content in T-2 and X-62 increased by 62.65% and 19.69%, respectively. In comparison to NaCl treatment, NaCl + G1~G4 treatments reduced the Na^+^ content in both cotton seedlings. The Na^+^ content in T-2 decreased by 17.83~21.86%, with NaCl+G4 treatment showing the greatest reduction. The Na^+^ content in X-62 decreased by 11.74~20.76%, with NaCl + G3 treatment showing the greatest reduction.

NaCl treatment significantly reduced the K^+^ content and the K^+^/Na^+^ ratio in T-2 and X-62 seedlings (*p* < 0.05). Compared to CK, under NaCl treatment, the K^+^ content in T-2 and X-62 decreased by 11.42% and 60.55%, and the K^+^/Na^+^ ratio decreased by 45.51% and 100.20%, respectively. In comparison to NaCl treatment, NaCl + G1~G4 treatments increased the K^+^ content and K^+^/Na^+^ ratio in both cotton seedlings. The K^+^ content in T-2 and X-62 increased by 2.53~12.89% and 14.10~39.16%, respectively, with NaCl + G3 treatment showing the greatest increase. The K^+^/Na^+^ ratio in T-2 increased by 20.44~28.08%, with NaCl + G2 treatment showing the greatest increase, while in X-62, the K^+^/Na^+^ ratio increased by 29.54~76.33%, with NaCl + G3 treatment showing the greatest increase.

### 2.5. ROS Production and Antioxidant Enzymes

**Figure 5:**
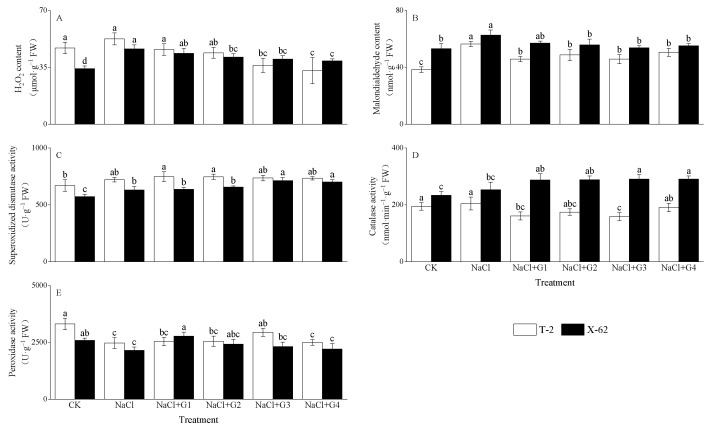
Effect of GABA on H_2_O_2_ (**A**), MDA (**B**), SOD (**C**), CAT (**D**), and POD (**E**) of cotton seedling leaves under salt stress. Different lowercase letters indicate a significant difference in the mean value of different treatments for the same cotton cultivar at the probability level of 0.05 (*p* < 0.05), determined by one-way analysis of variance (ANOVA) and Duncan’s post hoc test. The data are presented as means ± standard deviation (SD) calculated from three repetitions. T-2: Tahe 2; X-62: Xinluzhong 62. CK: 0 mM NaCl + 0 mM GABA; NaCl: 150 mM NaCl; NaCl + G1: 150 mM NaCl + 1 mM GABA; NaCl + G2: 150 mM NaCl + 2 mM GABA; NaCl + G3: 150 mM NaCl + 3 mM GABA; NaCl + G4: 150 mM NaCl + 4 mM GABA.

NaCl treatment increased the hydrogen peroxide (H_2_O_2_) and malondialdehyde (MDA) content in the leaves of Tahe 2 (T-2) and Xinluzhong 62 (X-62) seedlings (Figure 5A,B). Compared to CK, under NaCl treatment, the H_2_O_2_ content in T-2 and X-62 increased by 12.11% and 35.42%, respectively, with X-62 showing a significant difference from the CK (*p* < 0.05). The MDA content in T-2 and X-62 increased by 47.03% and 18.07%, respectively, and both showed significant differences from the CK (*p* < 0.05). In comparison to NaCl treatment, NaCl + G1~G4 treatments reduced the H_2_O_2_ and MDA content in T-2 and X-62. The reduction in H_2_O_2_ content in T-2 and X-62 ranged from 12.46% to 37.32% and from 5.72% to 15.79%, respectively, with NaCl + G4 treatment showing the greatest reduction. The reduction in MDA content in T-2 and X-62 ranged from 10.27% to 18.90% and from 8.89% to 14.31%, respectively, with NaCl + G3 treatment showing the greatest reduction.

NaCl treatment increased the activities of superoxide dismutase (SOD) and catalase (CAT) in the leaves of Tahe 2 (T-2) and Xinluzhong 62 (X-62) seedlings (Figure 5C,D). Compared to CK, under NaCl treatment, the SOD activity in T-2 and X-62 increased by 7.89% and 10.33%, respectively, and the CAT activity in T-2 and X-62 increased by 5.01% and 8.69%, respectively. In comparison to NaCl treatment, NaCl + G1~G4 treatments increased the SOD activity in T-2 and X-62. The increase in SOD activity in T-2 ranged from 1.67% to 3.63%, with NaCl + G1 treatment showing the greatest increase. The increase in SOD activity in X-62 ranged from 0.89% to 12.79%, with NaCl + G3 treatment showing the greatest increase. In comparison to NaCl treatment, NaCl + G1~G4 treatments decreased the CAT activity in T-2, with the greatest reduction (6.81% to 22.43%) observed with NaCl + G3 treatment. NaCl + G1~G4 treatments increased the CAT activity in X-62, with the greatest increase (13.43% to 14.49%) observed with NaCl + G3 treatment. Compared to CK, under NaCl treatment, the activities of peroxidase (POD) in the leaves of T-2 and X-62 seedlings decreased by 25.41% and 16.89%, respectively (Figure 5E). In comparison to NaCl treatment, NaCl + G1~G4 treatments increased the POD activity in T-2 and X-62. The increase in POD activity in T-2 ranged from 1.25% to 19.18%, with NaCl + G3 treatment showing the greatest increase. The increase in POD activity in X-62 ranged from 2.92% to 29.22%, with NaCl + G1 treatment showing the greatest increase.

### 2.6. Comprehensive Evaluation

**Figure 6:**
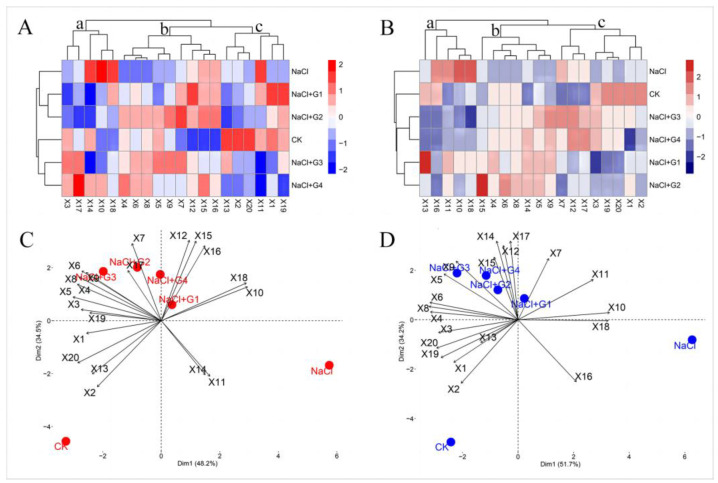
(**A**) Cluster analysis of indicators of cotton seedlings of Tahe 2, the indicators were grouped into three (a–c) distinct clusters. (**B**) cluster analysis of indicators of cotton seedlings of Xinluzhong 62, the indicators were grouped into three (a–c) distinct clusters. (**C**) principal component analysis of indicators of cotton seedlings of Tahe 2, (**D**) principal component analysis of indicators of cotton seedlings of Xinluzhong 62, bi-label plot. X1: germination rate, X2: germination index, X3: plant height, X4: aerial part fresh weight, X5: aerial part dry weight, X6: root fresh weight, X7: root dry weight, X8: total fresh weight, X9 total dry weight, X10: malondialdehyde, X11: H_2_O_2_, X12: superoxide dismutase, X13: peroxidase, X14: catalase, X15: proline, X16: Betaine, X17: Soluble sugars, X18: Na^+^, X19: K^+^, X20: K^+^/Na^+^ ratio. CK: 0 mM NaCl + 0 mM GABA; NaCl: 150 mM NaCl; NaCl + G1: 150 mM NaCl + 1 mM GABA; NaCl + G2: 150 mM NaCl + 2 mM GABA; NaCl + G3: 150 mM NaCl + 3 mM GABA; NaCl + G4: 150 mM NaCl + 4 mM GABA.

From Figure 6A,B, it can be observed that Tahe 2 (T-2) and Xinluzhong 62 (X-62) under different treatment conditions can be classified into three categories, labeled as a, b, and c. The two cultivars have different components in each category. For category a, the components for T-2 include X3, X17, X14, X10, and X18 (Figure 6A), while those for X-62 include X13, X16, X11, X10, and X18 (Figure 6B). Additionally, under different treatments, these two cultivars can be classified into two groups, and the components of these two groups also differ. This indicates cultivars in the alleviation effects of exogenous GABA on the indicators of the two cultivars. For T-2, CK, NaCl + G3, and NaCl + G4 are grouped together, while NaCl, NaCl + G1, and NaCl + G2 are in another group. As for X-62, CK, NaCl + G1, and NaCl + G2, as well as NaCl + G3 and NaCl + G4, are grouped together, with NaCl forming an independent group. The classification under different treatments demonstrates that exogenous GABA contributes to alleviating salt stress in both cotton cultivars, particularly in the case of X-62.

Using Principal Component Analysis (PCA) to further explore the correlations between treatments and cultivars, the results are shown in Figure 6. For Tahe 2 (Figure 6C), Dim1 explains 48.2% of all cultivars, Dim2 explains 34.5%, and together they account for 82.7%. For Xinluzhong 62 (Figure 6D), Dim1 explains 51.7% of all variables, Dim2 explains 34.2%, and together they account for 85.9%. It is noteworthy that NaCl for both varieties is located in the lower-right quadrant, CK is in the lower-left quadrant, NaCl + G2, NaCl + G3, and NaCl + G4 are in the upper-left quadrant, while NaCl + G1 is in the upper-right quadrant, suggesting potential similarities among them.

## 3. Discussion

### 3.1. Exogenous GABA Promotes Cotton Seed Germination and Seedling Growth

High salinity in saline soils was one of the crucial environmental factors inhibiting cotton seed germination [23]. Among these, NaCl was a primary salt contributing to soil salinization, and current research on plant salt stress mainly utilizes NaCl to simulate salt stress [2,3,17]. High concentrations of NaCl (150 mM) inhibit the activity of hydrolytic enzymes during the cotton seed germination phase, resulting in insufficient energy supply for seed germination, consequently reducing the index of cotton seed germination and significantly lowering the germination rate [3,7,8]. Although under the same salt stress conditions (150 mM NaCl) the germination rates of different cotton cultivars showed significant decreases, the extent of reduction varies. For instance, the reduction in the ‘Nongda Cotton 601’ cultivar was 50% [8], while the ‘GXM9’ cultivar experiences an 18% reduction [7]. In this study, the reduction rates for the ‘Tahe 2’ and ‘Xinluzhong 62’ cultivars were 20.34% and 32.14%, respectively. The differences in germination rates between cultivars may be attributed to variations in the salt tolerance genotypes. Under salt stress, compared to no GABA treatment, treatment with 1~4 mM GABA significantly increased the germination index of cotton seeds for both ‘Tahe 2’ and ‘Xinluzhong 62’ cultivars (Figure 1B) and improved the germination rate of seeds for both cultivars (Figure 1A). Similar reports suggested that exogenous GABA has a promotive effect on the germination capacity of seeds in wheat, maize, citrus, lettuce, ryegrass, and white clover [12,18,19,20,24]. The partial mechanisms through which exogenous GABA promotes seed germination may involve increasing the activity of α-amylase and β-amylase in seeds, thereby promoting starch metabolism to provide energy for cotton seed germination. Additionally, it might enhance the accumulation of dehydrin proteins during seed germination under salt stress and the expression levels of dehydrin-related genes [20].

Salt stress induced responses in plants such as a decrease in root-zone water potential and nutrient deficiency, leading to a series of survival strategies, including growth retardation, alterations in morphological characteristics, and biomass redistribution [3,4]. In this study, salt stress resulted in a reduction in plant height, aerial part fresh weight, aerial part dry weight, total fresh weight, and total dry weight of ‘Tahe 2’ and ‘Xinluzhong 62’ seedlings. However, it increased the root fresh weight and dry weight. Meanwhile, treatment with 1~4 mM GABA alleviated the growth inhibition of cotton seedlings under salt stress to varying degrees. It promoted an increase in plant height, aerial part fresh weight, aerial part dry weight, total fresh weight, and total dry weight. This effect was similar to the increase in plant height and biomass observed in cotton seedlings after foliar application of GABA [17]. Similar research results indicated that under salt stress, blackgrass and white clover seeds treated with GABA had higher plant height and biomass during the subsequent growth stage (seedling stage) [19,20]. Wu et al. [25] applied GABA treatment to the roots of tomato seedlings, resulting in an increased growth rate and biomass. This might be attributed to the enhancement of nitrogen assimilation-related enzyme activity by exogenous GABA, contributing to increased plant height and aerial part biomass in seedlings [14,19].

### 3.2. Exogenous GABA Regulates Osmoprotectants in Cotton Seedlings

Higher salt concentrations in the soil lead to soil osmotic pressure exceeding that of plant cells, making water absorption difficult for plants. This induced water loss, triggering osmotic stress and physiological drought within the plant [1,26]. To alleviate salt damage, plants accumulated osmoregulatory substances such as proline, betaine, and soluble sugars to maintain cell osmotic pressure and water status [2,3,4,8]. These osmoregulatory substances also serve other important functions, including regulating cell division, maintaining the stability of cell structures, and scavenging excess reactive oxygen species (ROS) [14,15,26,27]. It is noteworthy that different plant genotypes might exhibit distinct genetic traits in the production and accumulation of osmoregulatory substances, leading to varying responses to salt stress [27,28]. In this study, under the same salt stress conditions (150 mM NaCl), cotton seedlings of the ‘Tahe 2’ cultivar significantly increased the content of proline and betaine, while slightly decreasing the content of soluble sugars. For ‘Xinluzhong 62’ cotton seedlings, the content of proline, betaine, and soluble sugars all increased, with a significant impact on betaine. Under salt, compared to the no GABA treatment, exogenous GABA increased the content of proline and soluble sugars in both cotton cultivars under the same salt stress, consistent with previous research findings [14,29]. It is noteworthy that for the ‘Tahe 2’ cultivar, soluble sugars were more sensitive to GABA, while for ‘Xinluzhong 62’, proline was more sensitive to GABA. Limited research is available regarding the impact of exogenous GABA on plant betaine content under salt stress. Our study revealed a cultivar-specific response of betaine content in cotton seedlings to GABA. In comparison to the no GABA treatment, exogenous GABA had a smaller impact on betaine content in ‘Tahe 2’ seedlings under the same salt stress, while significantly reducing betaine content in ‘Xinluzhong 62’ cotton seedlings. This provided new insights into the effects of exogenous GABA on betaine content in different cultivars of the same crop. However, the connection between exogenous GABA and plant betaine still requires further clarification. Additionally, differential responses in osmoregulatory substances might have suggested the involvement of GABA in osmotic regulation in cotton seedlings. The addition of exogenous GABA induced the production of endogenous GABA in plants, and endogenous GABA possesses osmoregulatory functions [10,11,17].

### 3.3. Exogenous GABA Maintains Ion Homeostasis in Cotton Seedlings

As plants draw water and nutrients from the soil, a large amount of soluble salt enters the plant, leading to an excessive accumulation of Na^+^ within the plant. This can trigger the leakage of K^+^ from the cytoplasm, disrupting ion balance and, consequently, interfering with normal plant metabolic processes [27]. Similar observations were made in lettuce [24], chufa tubers [15], mungbean [14], tomato [25], and clover [20]. A key method to enhance plant salt tolerance is to maintain lower Na^+^ levels while sustaining higher K+ levels and K^+^/Na^+^ ratio [14]. Indeed, the K^+^/Na^+^ ratio was considered a crucial indicator for assessing salt tolerance in cotton [3,30]. In this study, excessive Na^+^ accumulation resulted in the yellowing of cotton seedling leaves (Figure 2), similar to symptoms of salt stress observed in rice [31]. Salt stress significantly decreased the K^+^ content and K^+^/Na^+^ ratio, consistent with the findings of Ma‘s study on cotton under the same salt treatment (150 mmol/L NaCl) [26]. While Ma ‘s study showed no significant impact of salt stress on the Na^+^ content, this study found a significant increase in the Na^+^ content in seedlings of ‘Tahe 2’ and ‘Xinluzhong 62’ under salt stress, possibly due to differences in cultivars and salt stress periods [26]. Under salt stress, compared to the no GABA treatment, treatment with 1~4 mM GABA reduced the Na^+^ content in cotton seedlings while increasing the K^+^ content and K^+^/Na^+^ ratio, in line with previous research results [9,14,17,20,32]. Particularly in this study, the K^+^/Na^+^ ratio in seedlings of ‘Xinlu Zhong 62’ decreased by 100.20% under salt stress, while treatment with 1~4 mM GABA increased the K^+^/Na^+^ ratio in cotton seedlings by 29.54% to 76.33%. Wu et al. [25] reduced the root absorption of Na^+^ and its transport to leaves in tomato seedlings by applying exogenous GABA to the roots. This result might be attributed to exogenous GABA inducing an increase in endogenous GABA content in plants [33], activating H+-ATPase to better maintain membrane potential, reducing stress-induced K^+^ leakage in roots. Additionally, GABA can enhance the expression levels of the SOS1 and NHX1 genes in leaves, promoting Na^+^ exclusion to the apoplast and sequestering Na^+^ in vacuoles, thereby reducing Na^+^ content in the cytoplasm [34]. Therefore, we believe that applying exogenous GABA treatment to cotton seeds under salt stress contributes to the maintenance of ion balance in seedlings for subsequent growth, imparting a robust salt-tolerant phenotype. This was evidenced by the absence of leaf yellowing in cotton plants under salt stress.

### 3.4. Exogenous GABA Alleviates Oxidative Stress in Cotton Seedlings

Salt stress induced osmotic stress and ion toxicity in plant cells, leading to the accumulation of ROS, such as hydrogen peroxide (H_2_O_2_), superoxide radicals (O^2−^), and hydroxyl radicals (OH^−^). This stress also induced lipid peroxidation of the cell membrane, resulting in a significant increase in malondialdehyde (MDA) content and ultimately disrupting normal cellular functions [1,2,10,13,15,27]. In this study, salt stress led to an increase in H_2_O_2_ content and a concomitant increase in MDA content in the leaves of ‘Tahe 2’ and ‘Xinluzhong 62’ seedlings. This aligned with previous observations of salt-induced oxidative stress in different plants [4,14,20,35,36]. Under salt stress, compared to the no GABA treatment, treatment with 1~4 mM GABA reduced the H_2_O_2_ and malondialdehyde (MDA) content in cotton seedlings, consistent with previous research findings [14,15,20,35,36]. Recent studies suggest that exogenous GABA could inhibit the production of H_2_O_2_ under salt stress in plants and regulate the expression of genes involved in H_2_O_2_ production [10].

To cope with oxidative damage caused by stress, plants evolved a series of crucial antioxidant enzymes, including superoxide dismutase (SOD), catalase (CAT), peroxidase (POD), glutathione peroxidase (GPX), glutathione reductase (GR), and ascorbate peroxidase (APX). These enzymes played a vital role in maintaining oxidative balance within the cell [2,3,4,11,14,17]. SOD acted as the first line of defense in the plant antioxidant system, converting superoxide molecules into oxygen and H_2_O_2_. Subsequently, CAT, APX, and POD converted H_2_O_2_ into water and oxygen. The collaborative action of these enzymes helps eliminate malondialdehyde (MDA) produced by lipid peroxidation, thereby protecting cell membrane structure [15,27]. Under salt stress, more energy in plants might have been allocated to upregulate antioxidant mechanisms to counteract damage caused by ROS, rather than to organ growth, resulting in reduced biomass [14]. In this study, salt stress led to a significant increase in SOD and CAT activity (Figure 5C,D), indicating their crucial role in regulating salt resistance in cotton seedlings. However, salt stress significantly decreased POD activity (Figure 5E). The reason for this result might have been that under high-salt (150 mM NaCl) conditions, the K^+^/Na^+^ ratio was significantly lower than that in the control treatment (Figure 4), leading to the accumulation of a large amount of Na^+^ in plant cells. These additional Na^+^ may have interacted with the POD enzyme through a certain process (salting-out) [37], resulting in a decrease in enzyme activity. This was further manifested as a reduction in POD activity in this experiment. In addition, the excessive production of reactive oxygen species might have led to enzyme inactivation, or interference with the synthesis of the enzyme or its subunits might have also contributed to this phenomenon. This result could be due to enzyme inactivation caused by excessive production of ROS or interference with the synthesis of the enzyme or its subunits [38,39]. For example, under the same salt stress conditions (150 mM NaCl), cotton seedlings of ‘Nongda Cotton 601’ showed a decrease in POD [8]. Under salt stress, exogenous GABA treatment increased the activities of SOD and POD in cotton seedlings. This alignment with previous research findings [14,35] further indicated that GABA could alleviate oxidative damage caused by salt stress through direct or indirect mechanisms [10,11]. Interestingly, the impact of exogenous GABA on CAT activity in cotton seedlings of ‘Tahe 2’ and ‘Xinluzhong 62’ under salt stress was the opposite. We believe the reason for this phenomenon may be due to the variation in CAT activity response among different varieties [40]. Based on our data, it may also be attributed to the differences in betaine content in the two varieties in response to exogenous GABA. However, further experiments are needed to explore the effects of exogenous GABA on betaine content and CAT activity in cotton of different varieties.

## 4. Materials and Methods

### 4.1. Experimental Location and Materials

The experiment was conducted from March to May 2023 in the climate chamber of the Baicheng Agricultural Experimental Station, Xinjiang Academy of Agricultural Sciences (81°87′ N, 41°80′ E). The artificial climate chamber conditions were set at 25 °C, 70% relative humidity, 12 h of light/12 h of darkness, with a light intensity of 284 μmol·m^−2^·s^−1^. The tested materials, upland cotton cultivars ‘Tahe 2’ and ‘Xinluzhong 62’, were provided by Tahe Seed Industry (Alar, Xinjiang). The seeds of ‘Tahe 2’ were deep brown, with a thousand-grain weight of 87.55 g, while the seeds of ‘Xinluzhong 62’ were light brown, with a thousand-grain weight of 90.90 g. The germination box dimensions were 19 cm in length, 13 cm in width, and 12 cm in height. Sodium chloride was obtained from Tianjin Xinbote Chemical Co., Ltd. (Tianjin, China). Gamma-aminobutyric acid (GABA) was obtained from Shanghai McLean Biochemical Technology Co., Ltd. (Shanghai, China). In this study, all chemical reagents were of analytical grade.

### 4.2. Experimental Design

Select full and uniformly sized cotton seeds for the experiment. Treat the seeds with a 0.4% potassium permanganate solution for 15 min, followed by rinsing five times with distilled water. On a clean bench at a temperature of 25 °C, spread the sterilized seeds evenly and air-dry them for 24 h for later use.

Experimental setup with 6 treatments: CK: 0 mM NaCl + 0 mM GABA; NaCl: 150 mM NaCl; NaCl + G1: 150 mM NaCl + 1 mM GABA; NaCl + G2: 150 mM NaCl + 2 mM GABA; NaCl + G3: 150 mM NaCl + 3 mM GABA; NaCl + G4: 150 mM NaCl + 4 mM GABA. Each treatment is replicated 3 times, with 20 seeds per replicate.

In each germination box, 250 g of vermiculite was spread, and 350 mL of the treatment solution (previously determined in a preliminary experiment to the point where squeezing the vermiculite after adding the solution does not cause water droplets) was added. It was covered with plastic wrap and left standing for 12 h. Afterward, 4 × 5 small holes were uniformly arranged in the cultivation box, with a depth of 1.5 cm and equal spacing between holes to ensure that the seeds did not touch each other. After sowing, the surface of the vermiculite was flattened. During the cultivation period, distilled water was supplemented as needed to maintain 60~80% of the water holding capacity of the vermiculite, and no fertilization was applied. The number of germinated seeds was observed and recorded daily during the germination period. Destructive sampling was performed 31 d after sowing to determine relevant indicators.

### 4.3. Measurement of Growth Parameters

Record the daily number of germinated seeds (considering visible seedlings), and stop recording on day 8. Calculate the Germination index (GI) [41] and germination rate (GR) [42] based on the following formulas.

(1) $$GI(\%)=\sum \left(\frac{G_{i}}{D_{i}}\right)$$

(2) $$GR\left(\%\right)=\left(\frac{N_{8}}{N}\right)\times 100\%$$

G_i_ is the germination rate on the i d after sowing, D_i_ is the i d germination of germination, N_8_ is the number of normal germinated seeds on the 8th d after sowing, and N is the total number of seeds tested.

Select three representative cotton seedlings from each treatment, and capture photographs using a digital camera (Canon EOS 90D, Tokyo, Japan). Using scissors, cut three leaves from each cotton plant within each treatment, ensuring the samples have the same weight. Wrap the samples in aluminum foil and store them in a foam box containing liquid nitrogen for subsequent enzyme activity measurements. Next, select three uniform seedlings from each group, thoroughly rinse the roots with distilled water, blot the surface moisture with filter paper, and measure the plant height with a ruler. Plant height is defined as the distance from the base of the stem to the growing point [43]. Use scissors to separate the seedlings into aerial part and root parts, recording the fresh weight of each. Then, place them in parchment paper bags, kill them in an oven at 105 °C for 30 min, followed by drying at 80 °C for 72 h, and record the dry weight [4].

### 4.4. Determination of Osmoprotectant Content

To determine the proline content, 0.5 g of leaves was first taken and mixed with 5 mL of a 3% sulfosalicylic acid solution, ensuring that the process was carried out under cold conditions. The mixing was performed using a mortar and pestle, following the method described by Mahmud et al. [44]. Subsequently, we placed the mixture in a centrifuge and centrifuged it at 10,000× *g* for 12 min to obtain the supernatant. Next, we took 1 mL of the supernatant and mixed it thoroughly with 1 mL of acid ninhydrin and 1 mL of glacial acetic acid. The mixture was then incubated at 95 °C in a hot water bath for 10 min. After the incubation, the mixture solution was transferred to a clean test tube and placed in an ice-containing box for proper cooling. Following that, 2 mL of toluene were added to the cooled solution, and the solution was thoroughly vortexed. Finally, we recorded the absorbance of the toluene containing the chromophore spectrophotometrically at a wavelength of 520 nm, and the proline content was estimated using a standard curve generated from known concentrations.

To determine the content of soluble sugars, the method described by Li et al. [8] was followed. A total of 1 g of leaves was placed in a 10 mL centrifuge tube, and 9 mL of distilled water was added to each tube. The mixture was ground into a homogenous slurry. A 1-milliliter aliquot of the supernatant was taken and mixed with 5 mL of sulfuric acid-anthrone reagent, followed by boiling for 10 min and subsequent cooling. The calorimetric method was employed to measure absorbance at 630 nm, and the soluble sugar content was calculated based on these data.

To determine the content of betaine, the method described by Zhang et al. [45] was followed. Leaves were dried at 80 °C for 24 h and finely ground. One milliliter of 80% methanol was mixed with the dried and ground sample (0.2 g), and the mixture was shaken for half an hour in a 60 °C water bath. After shaking, the mixture was centrifugated at 11,000× *g* for a quarter to harvest the extraction at 25 °C. Subsequently, 0.35 mL of saturated carnitine salt solution was added to the extraction solution (0.25 mL) and allowed to react for 4 h at 2 °C. After centrifuging at 10,000× *g* for 15 min at 25 °C, the supernatant was discarded, and the precipitate was washed with 0.3mL of 99% ether. The precipitate was then dissolved in 70% acetone (1 mL). Finally, a spectrophotometer was used to measure the absorbance at 525 nm.

### 4.5. Determination of Ion Content

To determine the Na^+^ and K^+^ content, the method described by Desoky et al. [46] was followed. First, the whole seedlings were rinsed with distilled water, dried at 80 °C for 72 h, and then finely ground. Dried leaves (0.1 g) were digested with a mixture of perchloric acid (2 mL, 80%) and concentrated H_2_SO_4_ (10 mL) for 12 h. Each sample was diluted to 100 mL with distilled water and determined for Na^+^ content and K^+^ content using a flame spectrophotometer. Na^+^ content and K^+^ content are expressed in mg·g^−1^ dry weight. K^+^/Na^+^ is the ratio determined by dividing the K^+^ content by the Na^+^ content.

### 4.6. Determination of H_2_O_2_ and Malondialdehyde Content

To determine the content of H_2_O_2_, the method described by Liu et al. [47] was followed. A total of 0.5 g of leaves was ground with pre-chilled acetone. The homogenate was centrifuged at a low temperature for 20 min, and 1 mL of supernatant was mixed with 0.1 mL of 10% TiCl_4_ and 0.2 mL of NH_3_·H_2_O. After reacting for 5 min, this was further centrifuged at 12,000× *g* and a temperature of 4 °C for 15 min. The precipitate was collected, 3 mL of 2 M H_2_SO_4_ was added, and the absorbance was measured at 415 nm.

To determine the content of malondialdehyde (MDA), the method described by Keya et al. [4] was followed. Initially, 0.5 g of leaves was taken and incubated with 5 mL of thiobarbituric acid (TBA) at 95 °C in a water bath. After 30 min of incubation, the mixture was transferred to a new transparent test tube and appropriately cooled in an ice-containing box. Subsequently, centrifugation was carried out at 10,000× *g* for 10 min at 25 °C. Next, 200 μL of the supernatant was pipetted into a micro quartz cuvette or a 96-well plate, and the absorbance at 532 nm and 600 nm was measured using a UV-Visible spectrophotometer. Finally, the content of MDA was estimated by calculating the difference in absorbance between 532 nm and 600 nm, allowing for the determination of the degree of lipid peroxidation in leaves.

### 4.7. Antioxidant Enzyme Activity Determination

To analyze antioxidant enzymes, we used 0.5 g of leaves. Firstly, fresh leaves were ground in a cold water bath and mixed with a 0.05 mol/L phosphate buffer saline (pH 7.4). Subsequently, they were centrifuged at 8000× *g* for 10 min at 4 °C. The resulting supernatant was used for the subsequent analysis of antioxidant substance content.

To determine the activity of superoxide dismutase (SOD), peroxidase (POD), and catalase (CAT), we utilized specific assay kits, namely, SOD-1-W, POD-1-Y, and CAT-1-Y, respectively. These assay kits were provided by Suzhou Keming Biotechnology Co., Ltd. (Suzhou, China).

### 4.8. Statistical Analysis

Data processing was conducted using Excel 2019, followed by an analysis of variance (ANOVA) and Duncan’s test for significance differences (*p* < 0.05) using SPSS 26. Line charts and bar graphs were created using Origin 2018. Hierarchical clustering and principal component analysis were performed using the “pheatmap” and “devtools” packages within R (v.4.3.1 for Windows) and RStudio IDE (2 June 2023).

## 5. Conclusions

Under salt stress, exogenous GABA treatment promoted seed germination in both cotton cultivars and effectively alleviated the adverse effects of salt stress on cotton seedling growth, including plant height, biomass, and leaf phenotype. Exogenous GABA treatment induced the accumulation of proline and soluble sugars in cotton seedlings while increasing the activity of antioxidant enzymes (SOD and POD), thereby slowing down the production of H_2_O_2_ and MDA resulting from oxidative damage. Additionally, exogenous GABA helped maintain K^+^/Na^+^ homeostasis. However, it is important to note that different cotton cultivars may exhibit varying responses to salt stress, and thus, the effectiveness of exogenous GABA in mitigating salt-induced damage may differ. These findings provide a foundation for a deeper understanding of the growth, biochemical, and physiological mechanisms by which GABA enhances salt tolerance in different cotton cultivars. Nevertheless, the molecular mechanisms of GABA-mediated salt tolerance in cotton still require further investigation.

## Figures and Tables

**Table 1:** Effects of GABA on plant height and biomass of cotton seedlings under salt stress.

Cultivar	Treatment	Plant Height (cm)	Aerial Part		Root		Total Fresh Weight (g·seedling^−1^)	Total Dry Weight (g·seedling^−1^)
			Fresh Weight (g·seedling^−1^)	Dry Weight (g·seedling^−1^)	Fresh Weight (g·seedling^−1^)	Dry Weight (g·seedling^−1^)		
T-2	CK	6.60 ± 0.26 ab	0.71 ± 0.07 a	0.14 ± 0.01 ab	0.74 ± 0.08 a	0.06 ± 0.01 a	1.46 ± 0.05 a	0.21 ± 0.00 b
	NaCl	5.93 ± 0.55 b	0.53 ± 0.07 b	0.11 ± 0.01 c	0.58 ± 0.09 b	0.07 ± 0.02 a	1.11 ± 0.03 b	0.18 ± 0.00 c
	NaCl + G1	6.10 ± 0.46 ab	0.68 ± 0.07 a	0.13 ± 0.01 b	0.75 ± 0.07 a	0.08 ± 0.00 a	1.43 ± 0.11 a	0.21 ± 0.00 b
	NaCl + G2	6.33 ± 0.31 ab	0.74 ± 0.05 a	0.15 ± 0.01 a	0.82 ± 0.01 a	0.08 ± 0.00 a	1.56 ± 0.04 a	0.23 ± 0.01 a
	NaCl + G3	6.73 ± 0.21 a	0.73 ± 0.03 a	0.15 ± 0.01 a	0.82 ± 0.03 a	0.08 ± 0.00 a	1.55 ± 0.05 a	0.23 ± 0.00 a
	NaCl + G4	6.60 ± 0.26 ab	0.75 ± 0.09 a	0.14 ± 0.01 ab	0.80 ± 0.08 a	0.07 ± 0.01 a	1.56 ± 0.15 a	0.21 ± 0.02 b
X-62	CK	7.90 ± 0.30 a	0.70 ± 0.01 a	0.13 ± 0.01 b	0.70 ± 0.04 a	0.06 ± 0.00 c	1.40 ± 0.03 a	0.19 ± 0.01 b
	NaCl	5.93 ± 0.15 c	0.55 ± 0.02 b	0.11 ± 0.01 c	0.53 ± 0.03 b	0.07 ± 0.00 ab	1.07 ± 0.01 b	0.18 ± 0.01 b
	NaCl + G1	6.40 ± 0.20 bc	0.70 ± 0.02 a	0.15 ± 0.01 a	0.68 ± 0.02 a	0.07 ± 0.00 b	1.38 ± 0.03 a	0.22 ± 0.01 a
	NaCl + G2	6.83 ± 0.61 b	0.69 ± 0.03 a	0.15 ± 0.01 a	0.73 ± 0.02 a	0.07 ± 0.00 bc	1.42 ± 0.01 a	0.22 ± 0.01 a
	NaCl + G3	7.77 ± 0.23 a	0.69 ± 0.02 a	0.16 ± 0.01 a	0.72 ± 0.03 a	0.08 ± 0.00 a	1.41 ± 0.04 a	0.23 ± 0.01 a
	NaCl + G4	7.67 ± 0.21 a	0.70 ± 0.01 a	0.15 ± 0.01 ab	0.71 ± 0.01 a	0.07 ± 0.00 ab	1.40 ± 0.02 a	0.22 ± 0.01 a

Note: Different lowercase letters indicate a significant difference in the mean value of different treatments for the same cotton cultivar at the probability level of 0.05 (*p* < 0.05), determined by one-way analysis of variance (ANOVA) and Duncan’s post hoc test. The data are presented as means ± standard deviation (SD) calculated from three repetitions. T-2: Tahe 2; X-62: Xinluzhong 62. CK: 0 mM NaCl + 0 mM GABA; NaCl: 150 mM NaCl; NaCl + G1: 150 mM NaCl + 1 mM GABA; NaCl + G2: 150 mM NaCl + 2 mM GABA; NaCl + G3: 150 mM NaCl + 3 mM GABA; NaCl + G4: 150 mM NaCl + 4 mM GABA.

## Data Availability

The data presented in this study are available in the figures and tables provided in the manuscript.
